# Supplementary figures and images for: Learning to use past evidence in a sophisticated world model
Source: PLoS Comput Biol. 2019 Jun 24;15(6):e1007093. doi: 10.1371/journal.pcbi.1007093 (PMC6611652; doi:10.1371/journal.pcbi.1007093)

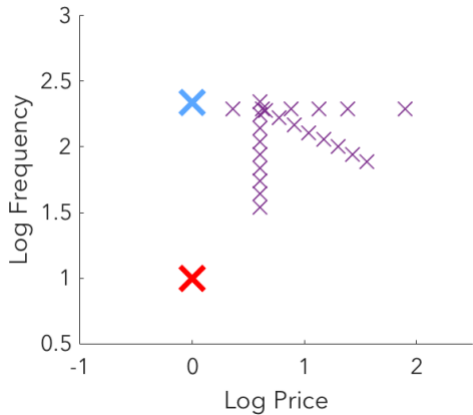

Supplement: S1 Fig — Frequencies and prices used during training for subject 1 are shown. The range of frequencies and prices is more limited than that employed in the full experiment. (PDF) [file pcbi.1007093.s001.pdf]

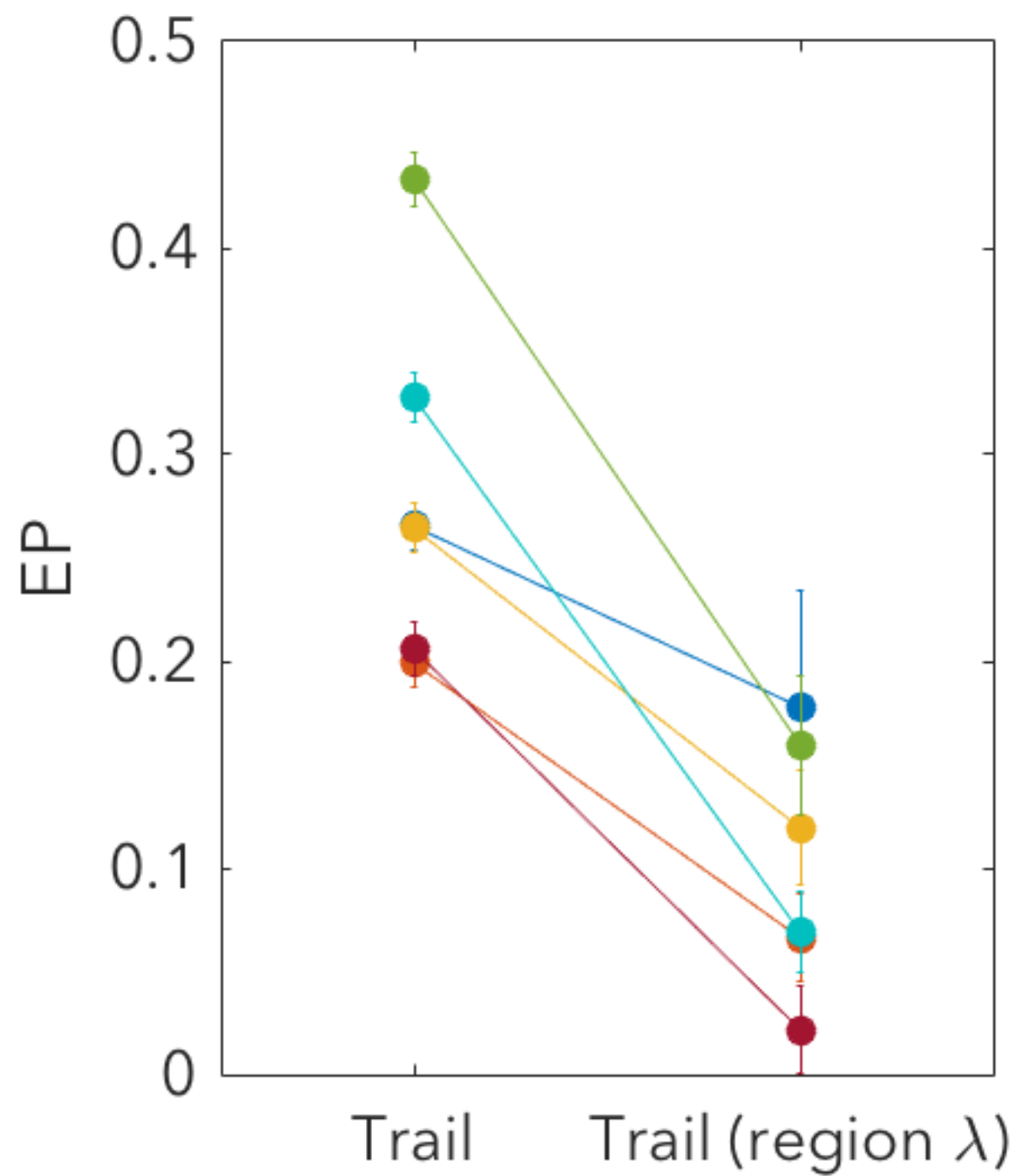

Supplement: S2 Fig — When trail trial responses are filtered such that only those with preceding test trials in region λ are included, a decrease in the EP is observed. (PDF) [file pcbi.1007093.s002.pdf]

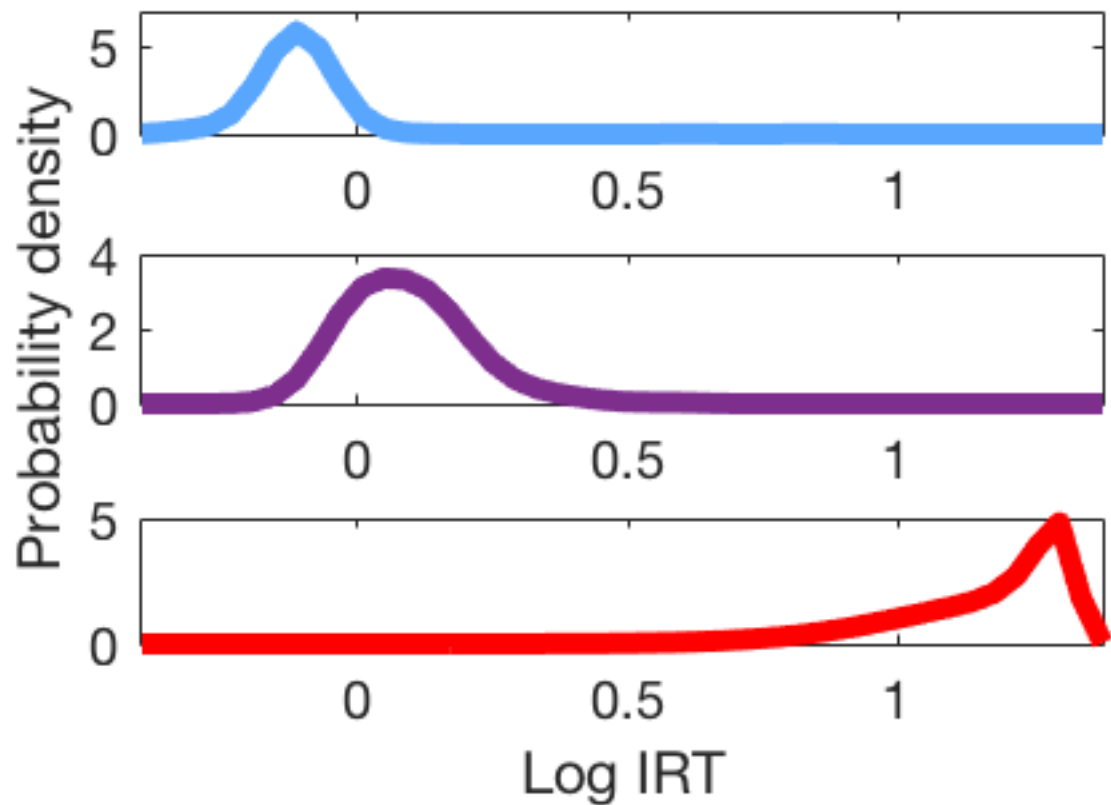

Supplement: S3 Fig — We evaluated the probability of the observed responses given certainty about the trial type by constructing kernel density estimates of the observed responses. For lead and test trials, which do not lead to confusion, the density estimate was based directly on the observed distributions. For trail trials, to account for confusion, we first filtered the trials such that only those with preceding test trials in region λ were included. (PDF) [file pcbi.1007093.s003.pdf]

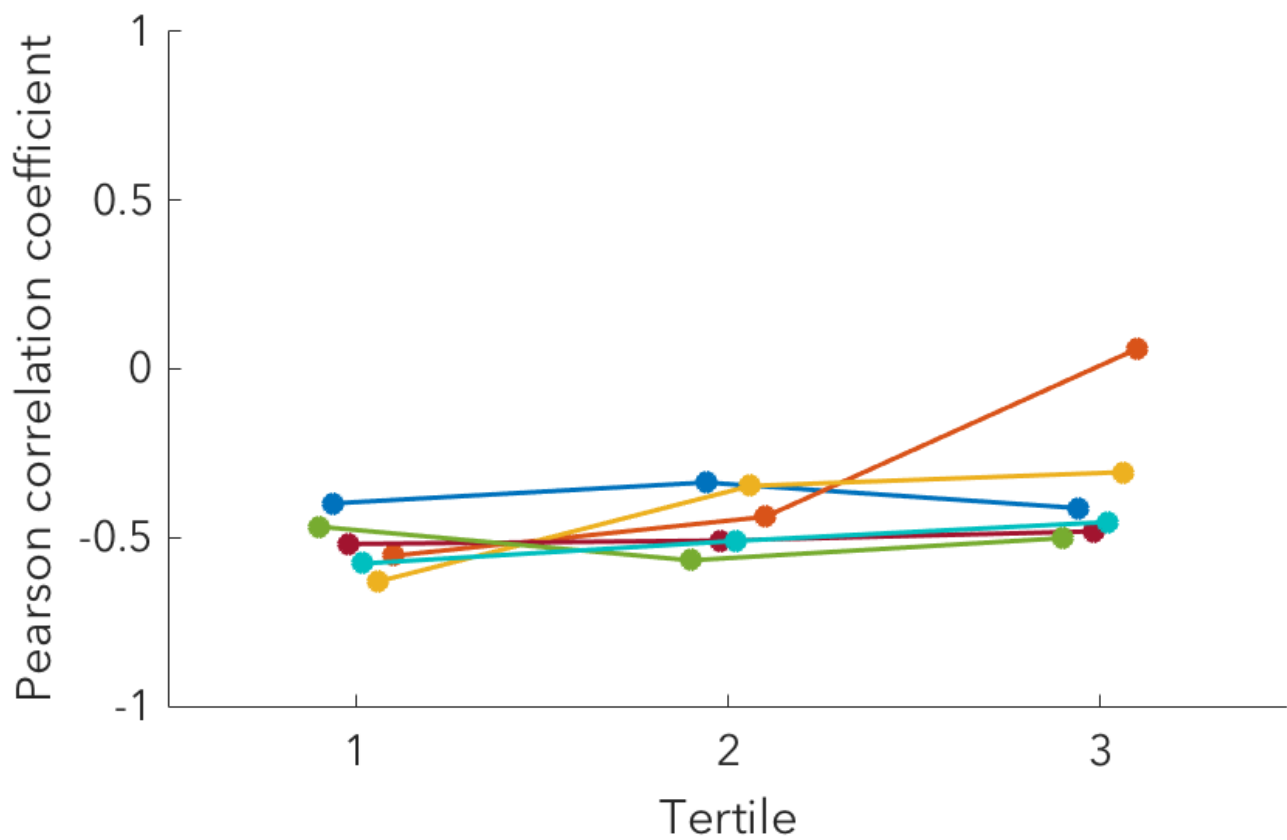

Supplement: S4 Fig — We determined the linear correlation between estimates of the parameters γ and σ by calculating the Pearson correlation coefficient. We calculated this coefficient separately for each subject and for each tertile and typically found a negative value between -0.3 and -0.7, indicating moderate anticorrelation. (PDF) [file pcbi.1007093.s004.pdf]
